# The Impact of Beta Blockers on Survival in Heart Transplant Recipients: Insights from the Zabrze HTx Registry

**DOI:** 10.1155/2020/5190248

**Published:** 2020-07-23

**Authors:** Grzegorz M. Kubiak, Radosław Kwieciński, Agnieszka Ciarka, Andrzej Tukiendorf, Piotr Przybyłowski, Tomasz Hrapkowicz, Michał O. Zembala

**Affiliations:** ^1^Department of Cardiac Vascular and Endovascular Surgery and Transplantology, Silesian Centre for Heart Diseases, Medical University of Silesia, Zabrze, Poland; ^2^Department of Cardiovascular Diseases, Catholic University of Leuven, Leuven, Belgium; ^3^Department of Public Health, Wrocław Medical University, Wrocław, Poland

## Abstract

**Introduction:**

The data assessing the impact of beta blocker (BB) medication on survival in patients after heart transplantation (HTx) are scarce and unequivocal; therefore, we investigated this population.

**Methods:**

We retrospectively analyzed the HTx Zabrze Registry of 380 consecutive patients who survived the 30-day postoperative period.

**Results:**

The percentage of patients from the entire cohort taking BBs was as follows: atenolol 24 (17%), bisoprolol 67 (49%), carvedilol 11 (8%), metoprolol 28 (20%), and nebivolol 8 (6%). The patients receiving BBs were older (56.94 ± 14.68 years vs. 52.70 ± 15.35 years, *p*=0.008) and experienced an onset of HTx earlier in years (11.65 ± 7.04 vs. 7.24 ± 5.78 *p* ≤ 0.001). They also had higher hematocrit (0.40 ± 0.05 vs. 0.39 ± 0.05, *p*=0.022) and red blood cells (4.63 (10^6^/*μ*l) ± 0.71 vs. 4.45 (10^6^/*μ*l) ± 0.68, *p*=0.015). Survival according to BB medication did not differ among the groups (*p*=0.655) (log-rank test). Univariate Cox proportional hazard regression analysis revealed that the following parameters were associated with unfavorable diagnosis: serum concentration of albumin (g/l) HR: 0.87, 95% CI (0.81–0.94), *p*=0.0004; fibrinogen (mg/dl) HR: 1.006, 95% CI (1.002–1.008), *p*=0.0017; and C-reactive protein (mg/l) HR: 1.014, 95% CI (1.004–1.023), *p*=0.0044.

**Conclusions:**

The use of BBs in our cohort of patients after HTx was not associated with survival benefits.

## 1. Introduction

Heart transplantation (HTx), which is the gold standard of treatment at the end stage of heart failure, is associated with denervation of the donor's heart. Since the reinnervation is reported in approximately fifty percent of cases and rarely affects the parasympathetic nervous system, the donors have increased heart rates [1, 2]. Physiological studies have shown that the exercise capacity of a denervated heart is determined by the increase in stroke volume through the Frank–Starling mechanism, and the raised heart rate (HR) contributes only later, at the peak of exercise [3]. The older studies have indicated that the use of nonselective beta blocker (BB) (propranolol) during physical activity resulted in reduced exercise tolerance and capacity [3, 4]. Therefore, BBs were advised to be used with caution; nonetheless, the early concerns about their use were recently challenged. Hence, a growing body of evidence shows that the elevated HR is associated with increased mortality and that the use of HR-decreasing medication in the selected group of patients may improve the short- and midterm results [5, 6]. Data on the use of BBs in HTx recipients in the clinical setting are scarce and unequivocal; therefore, we sought to investigate the impact of these drugs in the population of patients included in the institutional HTx Registry [7].

### 1.1. Aim

To test the hypothesis that HTx recipients who receive BBs during follow-up have a better prognosis.

## 2. Methods

**Figure 1:**
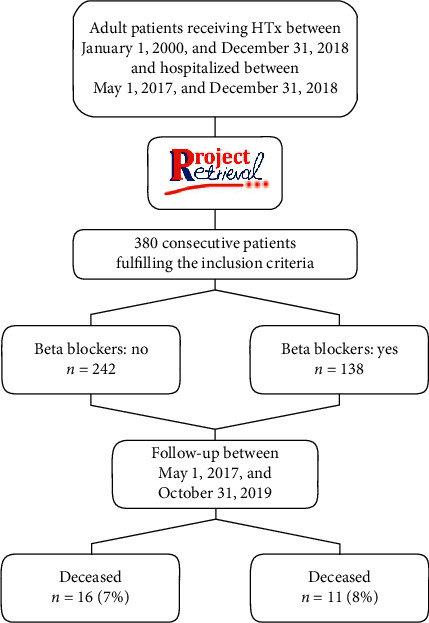
Flowchart of the study.

A total of 380 consecutive adult patients (≥18 years) receiving HTx at the Silesian Center of Heart Diseases between January 1, 2000, and December 31, 2018, who survived the in-hospital postoperative period and were hospitalized between May 1, 2017, and December 31, 2018, were retrospectively analyzed. The study complies with the Declaration of Helsinki and was approved by the local ethical committee which waived the need for informed consent (approval number ŚIL.KB.790.19). The follow-up between May 1, 2017, and October 31, 2019, was conducted in a typical manner in the outpatient clinic, using the phone contact or the digital information from the National Insurance System. The study flowchart is depicted in Figure 1. As the decision on the type of medication to implement was left at the physician's discretion, the authors did not interfere with this decision at any time point. The 4587 records of patients were collected using a computer software called the Retrieval Project, which was designed for this purpose. If the patient was eligible for the BB treatment, but it was discontinued for any reason and reported as such in a six- to twelve-month interval, the patient was described as not treated; this occurred in only three cases. The software was validated, and the data were manually verified to check if it was correctly retrieved.

### 2.1. Statistical Analysis

Distributions of the examined parameters were analyzed using the Shapiro–Wilk test. Categorical variables were expressed as *n* and percentage. Continuous variables were expressed as the mean ± standard deviation (SD). Linear variables with normal distribution were compared using Student's *t*-test. Variables with abnormal distribution were compared using the Mann–Whitney *U* test. Categorical variables were compared using the chi-square test. A Kaplan–Meier analysis was used to demonstrate the frequency of death due to any cause during the follow-up. Log-rank test was used to compare the survival curves based on BB use. Cox proportional hazard uni/multivariate regression approach was used to evaluate the risk of death. Independent predictors were presented as the hazard ratio (HR) with a confidence interval (CI). Differences between the values were considered statistically significant if *p* < 0.05. Analyses were performed using R statistical environment.

## 3. Results

The patients were more prone to receive BBs if they were diagnosed with supraventricular tachycardia 90 (65%) vs. 12 (5%) (*p* ≤ 0.001) and ventricular tachycardia 14 (10%) vs. 6 (2%) (*p* ≤ 0.001). BBs were also more frequently used in patients suffering from reduced left ventricular ejection fraction (LVEF) below fifty-five percent 40 (29%) vs. 30 (12%) (*p* ≤ 0.001). Notwithstanding, the use of BBs among patients diagnosed with hypertension or atrial fibrillation did not differ between the groups (114 (83%) vs. 188 (78%), *p* = ns, and 10 (7%) vs. 18 (7%), *p* = ns, respectively). Complex clinical characteristic (described as two or more indications) was common in both the BB and non-BB groups, 94 (68%) and 54 (22%), *p* ≤ 0.001. Dilated cardiomyopathy was less frequently reported as a reason for HTx in those patients who received BBs compared to those who did not 42 (30%) vs. 107 (44%), *p*=0.008. Compared to patients who were not on BBs, the patients receiving BBs were older (mean: 56.94 ± 14.68 years vs. 52.70 ± 15.35 years, *p*=0.008 ). They also had an earlier onset of HTx (mean: 11.65 ± 7.04 years vs. 7.24 ± 5.78 years, *p* ≤ 0.001) and higher initial HR (101.09 ± 12.26 (1/min) vs. 90.88 ± 12.32 (1/min), *p* ≤ 0.001). Other differences among the groups were statistically insignificant. The data are presented in Table 1.

**Figure 2:**
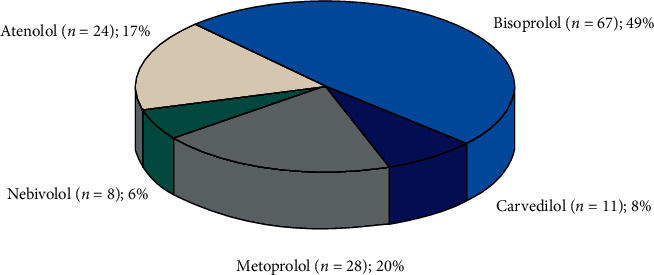
Each type of beta blocker used in the cohort.

Bisoprolol was used in 67 patients (49%), carvedilol in 11 patients (8%), metoprolol in 28 patients (20%), and nebivolol in 8 patients (6%). The data are depicted in Figure 2.

The patients receiving BBs had higher hematocrit and red blood cells than the non-BB patients (0.40 ± 0.05 vs. 0.39 ± 0.05, *p*=0.022 and 4.63 (10^6^/*μ*l) ± 0.71 vs. 4.45 (10^6^/*μ*l) ± 0.68, *p*=0.015, respectively). They also had lower platelets: 201.73 (10^3^/*μ*l) ± 62.39 vs. 220.44 (10^3^/*μ*l) ± 76.77, *p*=0.024. Additionally, the patients receiving BBs had decreased level of tacrolimus 8.45 (ng/ml) ± 3.63 vs. 9.40 (ng/ml) ± 3.83, *p*=0.037, and cyclosporin 110.60 (ng/ml) ± 32.04 vs. 141.01 (ng/ml) ± 49.76, *p*=0.033. Other differences including differences in biochemical, hematological, and coagulation parameters were statistically insignificant. All data are shown in Table 2.

**Figure 3:**
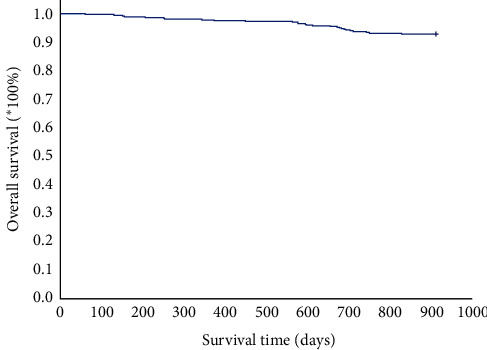
Survival probability of the patients fulfilling the inclusion criteria during the follow-up.

Over ninety percent of patients in the cohort completed the follow-up. The cumulative survival rate is depicted in Figure 3.

**Figure 4:**
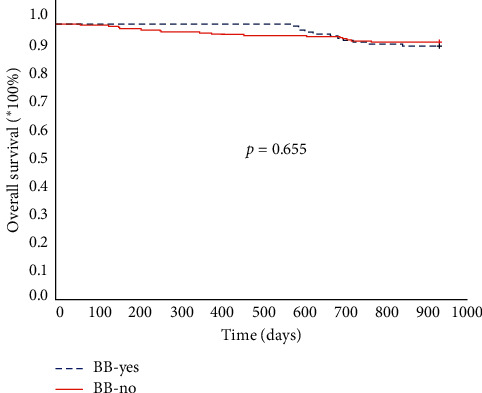
Kaplan–Meier survival curves depending on the beta blocker use during the follow-up.

Kaplan–Meier survival curves did not show statistically significant differences between those who received BBs and those who did not: *p*=0.655 (log-rank test). The Kaplan–Meier curves are shown in Figure 4.

Univariate Cox proportional hazard regression analysis revealed that the following parameters were associated with unfavorable prognosis: decreased albumin (g/l) HR: 0.87, 95% CI (0.81–0.94), *p*=0.0004; increased fibrinogen (mg/dl) HR: 1.006, 95% CI (1.002–1.008), *p*=0.0017; and increased CRP (mg/l) HR: 1.014, 95% CI (1.004–1.023), *p*=0.0044. Multivariate Cox proportional hazard regression model of survival did not show any significant predictors of death for any cause because the variables were too closely associated. The uni/multivariate Cox proportional hazard regression analysis is presented in Table 3.

## 4. Discussion

This paper has two major clinical implications. Firstly, the prognosis of the HTx patients who survived the in-hospital postoperative period in the eastern European country may not differ significantly from the western countries despite the substantial differences in the organization and funding of the healthcare systems [8, 9]. Secondly, we could not document the beneficial effect of BB administration on survival in the real-life clinical setting. Several aspects might have played a role. These include the older age of the patients receiving BBs compared to the control group (56.94 ± 14.68 vs. 52.70 ± 15.35, *p*=0.008); the more complex clinical profile of patients with more than one indication for the therapy (94 patients (68%) vs. 54 patients (22%), *p* ≤ 0.001); and the higher incidence of patients with the impairment of the systolic function of the heart (40 patients (29%) vs. 30 patients (12%), *p* ≤ 0.001).

In our cohort, BBs were mainly initiated in the treatment of supraventricular and ventricular tachycardia and in patients with impaired LVEF. Moreover, we reported a large number of patients receiving atenolol (*n* = 24, 17%) which has a lesser impact on hypertension, but not on heart rate, compared to bisoprolol [10–12]. The clinical practice at our center shows that BBs were introduced as a third or fourth line of drugs in the treatment of hypertension and frequently coadministered with calcium channel blockers (62 patients (45%) vs. 85 patients (35%), *p*=0.059); however, this difference was statistically insignificant. It is likely that using BBs in the cohort of older patients with higher incidence of comorbidities weakened or waived its potentially beneficial effect on survival [13, 14].

Ciarka et al. reported that BB treatment had beneficial effects on survival after HTx, which may be associated with its antiarrhythmic properties, especially given that it reduces the incidence of atrial fibrillation in the general population and after heart surgery [15–18]. In their study, patients receiving BB were older (52 ± 11 years vs. 49 ± 15 years, *p*=0.01) and had higher serum concentrations of total cholesterol (TC) (216 ± 53 vs. 204 ± 50 mg/dl, *p*=0.03) and low-density lipoprotein cholesterol (LDL-C) (120 ± 44 mg/dl vs. 107 ± 40 mg/dl, *p*=0.003). These results are in line with our observations. Patients receiving BBs in our study were also older (56.94 ± 14.68 years vs. 52.70 ± 15.35 years, *p*=0.008) and had higher concentrations of TC and LDL-C levels although without statistical significance (4.35 ± 1.34 vs. 4.25 ± 0.97 mmol/l and 2.21 ± 1.06 vs. 2.12 ± 0.84 mmol/l, *p* = ns, respectively). We observed that the patients receiving BBs had different blood morphology which was expressed by elevated red blood cells (RBC) (4.63 ± 0.71 vs. 4.45 ± 0.68 (10^6^/*μ*l), *p*=0.015), elevated hematocrit (0.40 ± 0.05 vs. 0.39 ± 0.05, *p*=0.022), and decreased platelets (201.73 ± 62.39 vs. 220.44 ± 76.77 (10^3^/*μ*l), *p*=0.024). It was reported that the hemoglobin concentration and hematocrit are raised in hypertensive patients [19, 20]. It has been proved in animal studies that the hemoglobin reduces the nitric oxide availability for the smooth muscle cells which implies vasoconstriction [21].

Despite the early controversies, there is a rising consensus that an increased heart rate stimulates coronary artery vasculopathy (CAV) and reduces survival after HTx [5–7, 22–26]. We found that the most advanced form of CAV, according to the ISHLT recommendations, occurred more often in patients receiving BBs than those who did not (13% vs. 7%, *p*=0.073), notwithstanding the difference was not statistically significant [27].

Nevertheless, it should be highlighted that the CAV, despite being present in half of the patients ten years after HTx, contributes only partially to the total mortality along with malignancies, infections, rejections, and renal insufficiencies [28, 29]. This contribution tends to be even lower in centers providing careful follow-up with annual invasive assessments and early revascularization of narrow stenoses [30]. Therefore, we cannot provide substantial evidence that the use of BBs in the patients without effective heart rate reduction has a beneficial effect on survival. Moreover, with the positive impact of diltiazem and ivabradine on cardiac remodeling and exercise tolerance, it would be interesting to compare their efficacy alone or in combination; thus, further investigations are warranted [31–33].

### 4.1. Limitations of the Study

The study is limited by the fact that only a part of the population of HTx patients has been analyzed due to technical reasons related to the automatic data acquisition. Moreover, we did not address the diastolic function of the heart and immunological agents which could have influenced the results.

## 5. Conclusions

The use of BB in our cohort of patients after HTx was not associated with improved survival. Further investigations including the use of other HR-decreasing agents like ivabradine or diltiazem, alone or in combination with BBs, are mandatory in the HTx patient population.

## Figures and Tables

**Table 1:** Patient characteristics.

	Beta blockers: no *n* = 242	Beta blockers: yes *n* = 138	*p*
Hypertension, *n* (%)	188 (78)	114 (83)	0.253
Atrial fibrillation, *n* (%)	18 (7)	10 (7)	0.945
Supraventricular tachycardia, *n* (%)	12 (5)	90 (65)	≤0.001
Ventricular tachycardia, *n* (%)	6 (2)	14 (10)	≤0.001
Reduced left ventricular ejection fraction <55%, *n* (%)	30 (12)	40 (29)	≤0.001
Two or more indications, *n* (%)	54 (22)	94 (68)	≤0.001
Age (years), mean ± SD	52.70 ± 15.35	56.94 ± 14.68	0.008
Women, *n* (%)	53 (22)	32 (23)	0.772
Dilated cardiomyopathy, *n* (%)	107 (44)	42 (30)	0.008
Ischemic cardiomyopathy, *n* (%)	83 (34)	50 (36)	0.704
Other etiologies of heart failure, *n* (%)	52 (21)	46 (33)	0.011
Time from HTx (years), mean ± SD	7.24 ± 5.78	11.65 ± 7.04	≤0.001
Left ventricular ejection fraction (%), mean ± SD	57.08 ± 4.49	55.89 ± 5.04	0.884
Heart rate (1/min), mean ± SD	90.88 ± 12.32	101.09 ± 12.26	≤0.001
Coronary artery vasculopathy grade 0, *n* (%)	139 (57)	82 (59)	0.706
Coronary artery vasculopathy grade 1, *n* (%)	54 (22)	24 (17)	0.253
Coronary artery vasculopathy grade 2, *n* (%)	30 (12)	16 (12)	0.818
Coronary artery vasculopathy grade 3, *n* (%)	18 (7)	18 (13)	0.073
Cause of death—any cause, *n* (%)	16 (7)	11 (8)	0.620
Cause of death—cardiovascular, *n* (%)	5 (2)	7 (5)	0.107
Cause of death—infection, *n* (%)	7 (3)	2 (1)	0.374
Cause of death—malignancy, *n* (%)	2 (1)	1 (1)	0.914
Cause of death—rejection, *n* (%)	1 (0)	1 (1)	0.687
Cause of death—other cause, *n* (%)	1 (0)	0 (0)	0.450
Start point of BBs after HTx (years), mean ± SD, (IQR)	n/a	5.76 ± 5.67 (0.75–9.50)	n/a
Time duration of BBs use (years), mean ± SD, (IQR)	n/a	5.91 ± 4.74 (2.57–7.77)	n/a
Atenolol daily dose, mean ± SD	n/a	43.75 ± 11.31	n/a
Bisoprolol daily dose (mg), mean ± SD	n/a	4.11 ± 2.88	n/a
Carvedilol daily dose (mg), mean ± SD	n/a	27.08 ± 18.40	n/a
Nebivolol daily dose (mg), mean ± SD	n/a	3.25 ± 1.68	n/a
Metoprolol daily dose (mg), mean ± SD	n/a	57.14 ± 52.05	n/a
Angiotensin-converting enzyme inhibitors	97 (40)	60 (43)	0.518
Angiotensin receptor blockers	42 (17)	16 (12)	0.133
Calcium channel blocker (CCB)	85 (35)	62 (45)	0.059
Dihydropyridine CCBs	83 (34)	61 (44)	0.056
Non-dihydropyridine CCBs	2 (1)	1 (1)	0.914
Statins	235 (97)	133 (96)	0.695
Acetylsalicylic acid	233 (96)	134 (97)	0.672
Ivabradine	0 (0)	0 (0)	n/a
Loop diuretics	67 (28)	42 (30)	0.569

SD: standard deviation; BBs: beta blockers; CCBs: calcium channel blockers; HTx: heart transplantation; IQR: interquartile range; n/a: not applicable; FU: follow-up.

**Table 2:** Laboratory findings.

		Beta blockers: no Mean ± SD *n* = 242	Beta blockers: yes Mean ± SD *n* = 138	*p*
Creatinine (*μ*mol/l)		125.63 ± 48.63	134.73 ± 64.66	0.597
Serum glucose (mmol/l)		6.18 ± 2.45	6.31 ± 1.79	0.622
Hemoglobin A1c (%)		5.50 ± 0.78	5.58 ± 0.87	0.489
White blood cells (10^3^/*μ*l)		7.75 ± 3.76	7.31 ± 2.54	0.464
Red blood cells (10^6^/*μ*l)		4.45 ± 0.68	4.63 ± 0.71	0.015
Platelets (10^3^/*μ*l)		220.44 ± 76.77	201.73 ± 62.39	0.024
Hemoglobin (mmol/l)		8.12 ± 1.25	8.29 ± 1.22	0.132
Hematocrit		0.39 ± 0.05	0.40 ± 0.05	0.022
C-reactive protein (mg/l)		8.09 ± 20.48	16.54 ± 33.81	0.131
Aspartate transaminase (IU/l)		24.70 ± 14.64	25.43 ± 18.11	0.693
Alanine aminotransferase (IU/l)		21.88 ± 15.67	23.01 ± 21.25	0.946
Total serum bilirubin (*μ*mol/l)		14.11 ± 10.89	13.38 ± 7.42	0.403
Total protein (g/l)		69.95 ± 6.62	69.68 ± 5.94	0.520
Albumin (g/l)		43.64 ± 4.64	42.82 ± 4.37	0.114
Gamma-glutamyl transpeptidase (IU/l)		88.02 ± 259.19	77.94 ± 140.54	0.694
Alkaline phosphatase (IU/l)		92.39 ± 48.75	106.84 ± 165.61	0.603
Creatine phosphokinase (IU/l)		124.01 ± 137.41	126.94 ± 149.25	0.687
Uric acid (*μ*mol/l)		402.29 ± 114.00	385.01 ± 99.02	0.337
Urea (mmol/l)		8.47 ± 4.20	8.98 ± 5.06	0.760
Cystatin (mg/L)		1.48 ± 0.69	1.62 ± 0.93	0.654
Cholesterol (mmol/l)		4.25 ± 0.97	4.35 ± 1.34	0.892
Low-density lipoprotein (mmol/l)		2.12 ± 0.84	2.21 ± 1.06	0.783
High-density lipoprotein (mmol/l)		1.37 ± 0.43	1.30 ± 0.43	0.225
Activated partial thromboplastin time (s)		34.01 ± 8.05	33.00 ± 5.78	0.132
International normalized ratio		1.08 ± 0.24	1.08 ± 0.27	0.467
Natrium (mmol/l)		139.94 ± 3.15	140.39 ± 2.71	0.264
Kalium (mmol/l)		4.66 ± 0.48	4.63 ± 0.52	0.641
Magnesium (mmol/l)		0.70 ± 0.13	0.72 ± 0.12	0.160
Fibrinogen (mg/dl)		362.77 ± 109.28	386.12 ± 134.60	0.177
Tacrolimus (ng/ml)		9.40 ± 3.83	8.45 ± 3.63	0.037
Mycophenolate mofetil (*μ*g/ml)		2.27 ± 1.11	2.24 ± 1.22	0.407
Cyclosporin (ng/ml)		141.01 ± 49.76	110.60 ± 32.04	0.033

SD: standard deviation.

**Table 3:** Uni/multivariate Cox regression analysis of risk factors.

Cox regression	Univariate			Multivariate		
Risk factor	HR	95% CI	*p*	HR	95% CI	*p*
Beta blocker: yes	0.54	(0.28–1.44)	0.2820	0.65	(0.22–1.93)	0.4342
CRP (mg/l)	1.014	(1.004–1.023)	0.0044	1.011	(0.999–1.023)	0.0667
Albumin (g/l)	0.87	(0.81–0.94)	0.0004	0.98	(0.86–1.12)	0.8006
Fibrinogen (mg/dl)	1.006	(1.002–1.008)	0.0017	1.005	(1.000–1.010)	0.0675

CI: confidence interval; CRP: C-reactive protein; HR: hazard ratio.

## Data Availability

The data used to support the findings of this study are restricted by the local ethical board (approval number ŚIL.KB.790.19) in order to protect patient privacy. Data are available from the corresponding author through gkubiak@sccs.pl for researchers who meet the criteria for access to confidential data.

## Additional Points

The free trial version of the ProjectRetrieval® software is available to download at the http://retrieval-project.com.pl. Its functionality is limited. Any potential users are kindly requested to contact the authors prior to the use of the software in order to set a consensus considering the donation and citation details.
